# Agonistic effect of polyunsaturated fatty acids (PUFAs) and its metabolites on brain-derived neurotrophic factor (BDNF) through molecular docking simulation

**DOI:** 10.1186/1476-511X-11-109

**Published:** 2012-09-04

**Authors:** Umashankar Vetrivel, Sathya Baarathi Ravichandran, Kaviarasan Kuppan, Jithu Mohanlal, Undurti Narasimha Das, Angayarkanni Narayanasamy

**Affiliations:** 1 Department of Bioinformatics, Vision Research Foundation, Chennai, 600 006, India; 2 Department of Biochemistry and Cell Biology, Vision Research Foundation, Chennai, 600 006, India; 3 School of Biotechnology, Jawaharlal Nehru Technological University, Kakinada, 533 003, India; 4 UND Life Sciences, 13800 Fairhill Road, #321, Shaker Heights, OH, 44120, USA; 5 Bio-Science Research Centre, Gayatri Vidya Parishad Engineering College, Visakhapatnam, 533 048, India

**Keywords:** BDNF, LXA4, 4-methyl catechol, TrkB, Diabetes

## Abstract

**Background:**

Brain-derived neurotrophic factor (BDNF) is a potent neurotrophic factor that is implicated in the regulation of food intake and body weight. Polyunsaturated fatty acids (PUFAs) localised in cell membranes have been shown to alter the levels of BDNF in the brain, suggesting that PUFAs and BDNF could have physical interaction with each other. To decipher the molecular mechanism through which PUFAs modulates BDNF’s activity, molecular docking was performed for BDNF with PUFAs and its metabolites, with 4-Methyl Catechol as a control.

**Results:**

Inferring from molecular docking studies, lipoxin A4 (LXA4), and a known anti-inflammatory bioactive metabolite derived from PUFAs, with a binding energy of −3.98 Kcal/mol and dissociation constant of 1.2mM showed highest binding affinity for BDNF in comparison to other PUFAs and metabolites considered in the study. Further, the residues Lys 18, Thr 20, Ala 21, Val 22, Phe 46, Glu 48, Lys 50, Lys 58, Thr 75, Gln 77, Arg 97 and Ile 98 form hot point motif, which on interaction enhances BDNF’s function.

**Conclusion:**

These results suggest that PUFAs and their metabolites especially, LXA4, modulate insulin resistance by establishing a physical interaction with BDNF. Similar interaction(s) was noted between BDNF and resolvins and protectins but were of lesser intensity compared to LXA4.

## Introduction

Neurotrophins protect and promote the growth and development of nerves [1]. They have potent effects on neuronal differentiation, survival, neurite outgrowth, synaptic formation, and plasticity [2,3]. Of all, BDNF and its receptor TrkB are the most abundantly expressed in hippocampus and cerebral cortex region of the brain [4]. BDNF plays a crucial role in controlling body weight and energy homeostasis [5]. Increased levels of BDNF reduce food consumption and maintain energy balance [6]. Both central and peripheral administration of BDNF decreased food intake, increased energy expenditure and ameliorated hyperinsulinaemia and hyperglycaemia in diabetic db/db mice [7-10].

TrkB activation by BDNF is essential for appetite regulation and energy homeostasis [8]. BDNF mediated TrkB signalling is an important downstream target for MC4R-mediated signalling which participates in the regulation of energy balance and feeding behaviour [6]. Mutations in the TrkB receptor resulted in hyperphagia and morbid obesity in humans and rodents. Conversely, peripheral or central stimulation of TrkB by its natural ligands BDNF or NT4 reduced body weight and food intake in mice, supporting the idea that TrkB is a key anorexigenic signal downstream of the melanocortin-4 receptor (MC4R) system [11].

Polyunsaturated fatty acids such as linoleic acid (LA), arachidonic acid (AA), α-linolenic acid (ALA), eicosapentaneoic acid (EPA) and docosahexaenoic acid are present in almost all cell membranes and regulate their (cell membrane) fluidity, receptor number and affinity of receptor to their respective hormones, peptides and growth factors [12-15]. In this context, it is interesting to note that dietary supplementation with omega-3 PUFAs reportedly normalizes BDNF levels which are reduced following brain injury [16]. Omega-3 enriched dietary supplement provides protection against reduced plasticity and impaired learning ability associated with brain injury in rats. High-fat diets, particularly those rich in saturated fats, adversely affect insulin action and alter homeostasis model assessment (HOMA) [17], which can be prevented by n-3 PUFAs rich in eicosapentaenoic acid (EPA) and docosahexaenoic acid (DHA) by reducing insulin resistance [18].

Furthermore, PUFAs are needed for synapse formation, neurite outgrowth and also have neuroprotective actions [19,20]. Nerve growth cones are highly enriched with AA-releasing phospholipases, which have been implicated in neurite outgrowth. Cell membrane expansion occurs through the fusion of transport organelles with plasma membrane, and syntaxin 3, a plasma membrane protein that is important in the growth of neurites, is a direct target for AA, DHA and other PUFAs. These (AA, DHA, and other PUFAs) polyunsaturated fatty acids but not saturated and monounsaturated fatty acids activate syntaxin 3.

SNAP25 (synaptosomal-associated protein of 25 kDa), a syntaxin partner implicated in neurite outgrowth, interacts with syntaxin 3 only in the presence of AA that allowed the formation of the binary syntaxin 3-SNAP 25 complex. AA stimulated syntaxin 3 to form the ternary SNARE complex (soluble N-ethylmaleimide-sensitive factor attachment protein receptor), which is needed for the fusion of plasmalemmal precursor vesicles into the cell surface membrane that leads to membrane fusion [21]. Thus, AA and DHA change the α-helical syntaxin structure to expose SNARE motif for immediate SNAP 25 engagement that facilitates neurite outgrowth. This could be one important mechanism by which AA, EPA and DHA are able to enhance neurogenesis and bring about their beneficial actions. In this context, it is interesting to note that BDNF and TrkB are expressed in the retinal ganglion cell layer and are able to influences the morphological differentiation of the cells and act as cell survival factors [22,23].

Since both BDNF and n-3 PUFAs levels are known to be altered in diabetes and several other neurological conditions, our current study aims to investigate the effect of PUFAs and their metabolites on BDNF binding towards the receptor, tropomyosin-related kinase-B (TrkB) and its function through molecular docking simulation. This will provide an insight on the bioactive conformation of the protein through simulation studies and the possible high affinity binding orientations of the ligands, thus paving way for understanding the modulating mechanism of PUFAs and its metabolites on BDNF’s affinity.

## Methods

### Molecular Dynamics simulation and Optimization

BDNF is a 247 amino acid protein, secreted in the extracellular space. The 3D (three dimensional) structure of the heterodimeric form of BDNF with Neurotrophin 3 **[PDBID: 1BND]** has been elucidated using X-ray crystallography at 2.30Å resolution [24]. To optimize and understand the conformational changes of the monomeric form of BDNF in the presence of explicit solvent, molecular dynamics simulation was performed using GROMACS (Groningen Machine for Chemical Simulations) 4.3.1 package [25]. The model system was solvated with three-point transferable intermolecular potential (TIP3P) molecules [26] in a cubic water box with the periodic boundary conditions set. To neutralize the system, eight CL − ions were added and subjected to energy minimization through Optimized Potentials for Liquid Simulations (OPLS) force field using the steepest descent integrator. After reaching the convergence limit, the BDNF in the system was subjected to position-restrains by keeping number of particles (N), pressure (P) and temperature (T) constant (NPT). Using Particle Mesh Ewald (PME) electrostatics method under NPT conditions, final MD simulation of 5,000,000 was performed for 10,000 ps (10 ns). Finally, the optimized structure was utilized for further docking studies.

### Small molecule optimization

3D Structural co-ordinates of 4-MC **[CID 9958]**, LXA4 **[CID 5280914]**, AA **[CID 444899]**, EPA **[CID 446284],** DHA **[CID 445580]**, RVE1 **[CID 10473088]** and NPD1 **[CID 16042541]** were retrieved from Pubchem database [27] and were geometry optimized using PRODRG server with full charges [28].

### Protein-Ligand docking

Molecular Docking simulation of the ligands with the monomeric form of BDNF was carried out using AutoDock 4.2 in combination with Lamarckian genetic algorithm (LGA) to find the optimal conformation of the ligand. The optimized 3D structure of BDNF was prepared by removing water molecules and CL- ions; polar hydrogens were added. Further, Kollman united atom charges and Gasteiger charges were added to the receptor and ligand, respectively. The atomic solvation parameters were assigned using the ADDSOL utility of Auto Dock 4.2. Flexibility of the ligands was assigned based on its torsional degrees of freedom through Autotors option, with the protein torsional angles were kept fixed throughout the process of docking simulation [29-31].

Grid box covering the complete surface of BDNF was constructed and used for molecular docking with a grid spacing of 0.514 Å and the number of points in each grid dimension set to 126 × 126 × 126. Further, Grid maps were generated for each atom within the ligands using Autogrid. Using LGA, molecular docking was carried out with default parameters, except for the number of GA runs which was set to 100, Maximum number of generations was set to 500 and maximum number of evaluations set to 250 000 [32].

Further, cluster analysis was performed by sorting the binding modes based on the binding energy within the specified RMSD threshold, which was set to 2Å. The optimal binding orientation of the ligand is the one with the lowest binding energies picked up from the cluster, which has maximum number of similar conformations. However, agonistic mode was screened based on the binding energy, dissociation constant and through interaction studies [33]. Hot point interactions mediating BDNF - ligand complex were analyzed and visualized using PDBsum [34] and PyMOL (http://www.pymol.org), respectively.

## Results

### Molecular Dynamics simulation of BDNF

MD simulation of the monomeric form of BDNF was analyzed to gain a better insight into the stability and dynamics nature of the molecule. Initial analysis was performed to ascertain the quality of MD simulation performed. The quality checks involves convergence of thermodynamic parameters: temperature, potential and kinetic energy. Analyzing the graphs, convergence in all these parameters were observed, which is suggestive of system’s equilibrium.

**Figure 1:**
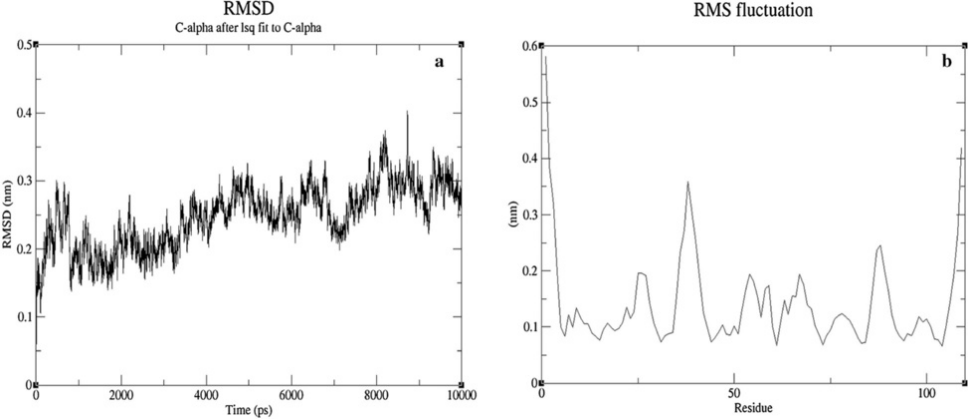
Inferring the structural stability of BDNF through RMSD (a) and RMSF (b).

Further, Systems equilibrium in terms of structure was inspected by comparing the Root Mean Square Deviation (RMSD) of the C-alpha with respect to the starting structure. Convergence of RMSD was inferred from the graph towards the process of simulation (Figure 1a). Root Mean Square Fluctuations (RMSF) was examined to infer the flexibility in the residues during the course of simulation (Figure 1b). These structural level analyses were indicative of the system reaching equilibrium. And, the final optimized monomeric form of BDNF was taken for further docking analysis.

### Molecular Docking studies with a known reference enhancer, 4-methyl catechol

**Figure 2:**
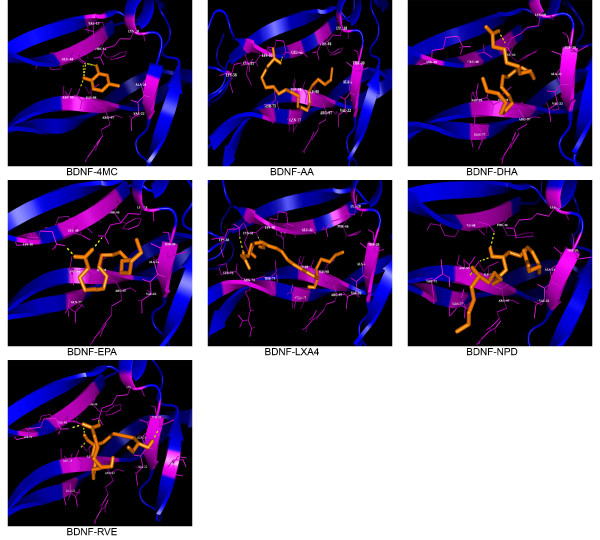
**Molecular interactions observed between BDNF and Ligands.** Interactions observed between ligand (Blue) and BDNF (RED) with hydrogen bonded interactions represented by YELLOW dashes.

Atsumi Nitta et al. proposed the stimulating effects of 4-MC on the synthesis of BDNF in neurons and astrocyte cells [35]. However, the agonistic binding mode of 4-MC on BDNF has not been studied. Therefore, molecular docking studies have been performed for BDNF with 4-MC. Among 69 conformers in Cluster1, the one with a lowest binding energies of −4.36Kcal/mol and dissociation constant of 635.36 μM was considered for the further analysis (Table 1). Inferring from interaction analysis, Tyr47, Glu48 and Asp99 showed hydrogen bond interactions; wherein Val 13, Lys 18, Ala 21, Val 22, Phe 46, Arg 97 and Ile 98 showed non-bonded interactions with 4-MC (Figure 2) (Table 1) and it could be a probable hot point residues which on interaction stimulates BDNF activity.

**Table 1:** Molecular interaction analysis of BDNF with the endogenous agonists and the control, 4-MC

**Ligands**	**Binding energy (kcal/mol)**	**Dissociation constant(mM)**	**Bonded and non-bonded interactions**
4-MC	-4.36	0.64	Val13, Lys18, Ala21, Val22, Phe46, Tyr47, Glu48, Arg97, Ile98 and Asp99
LXA4	-3.98	1.2	Thr 20, Ala 21, Phe 46, Lys 50, Cys 51, Lys 58,Cys 73, Arg 74, Thr 75, Gln 77,Arg 97, Ile 98 and Asp 99
EPA	-3.67	2.06	Lys 18, Thr 20, Ala 21, Val 22, Phe 46, Glu 48, Lys 50,Arg 97, Ile 98 and Asp 99
AA	-2.9	7.53	Lys 18, Thr 20, Ala 21, Val 22, Phe 46, Glu 48, Lys 50, Lys 58, Thr 75, Gln 77,Arg 97 and Ile 98
DHA	-2.89	7.66	Lys 18(3.05), Thr 20, Ala 21, Val 22,Phe 46, Glu 48, Gln 77, Arg 97, Ile 98 and Asp 99
RVE1	-2.51	14.39	Lys 18, Thr 20, Ala 21,Val 22, Thr 28, Phe 46, Glu 48,Lys50, Gln 77, Arg 97, Ile 98 and Asp 99.
NPD1	-2.1	28	Lys 18, Thr 20, Ala 21, Val 22, Glu 48, Thr 75, Gln 77, Arg 97, Ile 98 and Asp 99.

Residues involved in Hydrogen bonding interactions are bolded.

### Molecular Docking studies with PUFAs

The binding conformation of PUFAs such as DHA, EPA and AA has been inferred through molecular docking studies. Among the PUFAs considered in the study, EPA, an omega-3 fatty acid with a binding energy of −3.67 Kcal/mol and dissociation constant of 2.06mM showed highest binding affinity for BDNF by forming a hydrogen bond interaction with Lys 18 and Lys 50; non – bonded interactions with Thr 20, Ala 21, Val 22, Phe 46, Glu 48, Arg 97, Ile 98 and Asp 99. However, DHA and AA also showed relatively higher binding affinity than the metabolites with a binding energy of −2.9 Kcal/mol and −2.89 Kcal/mol, respectively (Table 1) (Figure 2).

### Docking interaction analysis of BDNF with metabolites REV1, NPD1 and LXA4

Molecular docking studies were performed to infer the mechanism through which metabolites of PUFAs mediates BDNFs activity. LXA4, an endogenously synthesized nonclassic eicosanoid, showed highest binding affinity for BDNF in comparison to the PUFAs and metabolites considered in the study with a binding energy of −3.98 Kcal/mol and dissociation constant of 1.2 mM. And, LXA4 established a network of hydrogen bond interactions with Cys 51 and Lys 58; hydrophobic interaction with Thr 20, Ala 21, Phe 46, Lys 50, Cys 73, Arg 74, Thr 75, Gln 77,Arg 97, Ile 98 and Asp 99 of BDNF. Wherein, NPD1 and REV1 showed comparatively lower binding affinity for BDNF with the binding energy of −2.51 Kcal/mol and −2.1 Kcal/mol, respectively (Table 1) (Figure 2).

**Figure 3:**
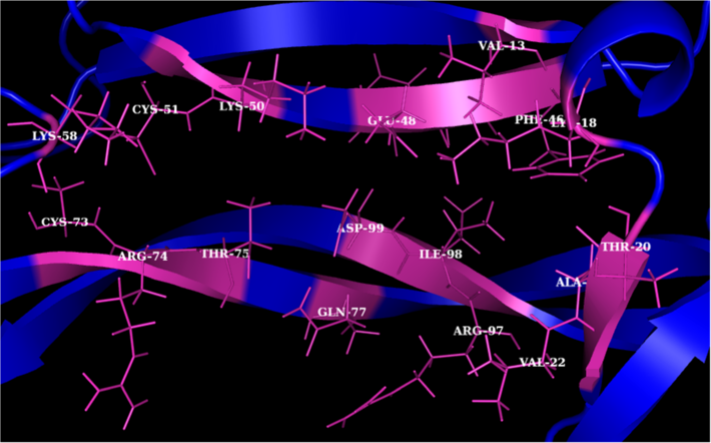
Hot Point residues (represented as sticks in magenta) of BDNF (blue).

Inspecting at the interacting residues, consensus in the residues mediating interaction with PUFAs and its metabolites was observed. Further, same residues were found to establish interaction with 4-MC and, these residues involve Lys 18, Thr 20, Ala 21, Val 22, Phe 46, Glu 48, Lys 50, Lys 58, Thr 75, Gln 77, Arg 97 and Ile 98. Thus, suggestive of these residues forming a probable hot point motif mediating the BDNFs activity (Figure 3).

## Discussion

Molecular docking studies with the optimized model of BDNF elicited the potential binding modes of agonists. The active binding mode of the catechol compound, 4-MC has been inferred through molecular docking studies. Thus confirming the 4-MC’s enhancing effect on BDNF mRNA expression and secretion. However, the inability of 4-MC to cross the Blood Brain Barrier limits it therapeutic efficacy, which in turn requires novel agonist for BDNF neurotrophic activity.

In case of endogenous agonists considered in the study, LXA4 showed highest binding affinity for BDNF by forming network of bonded and non- bonded interactions with Thr 20, Ala 21, Phe 46, Lys 50, Cys 51, Lys 58, Cys 73, Arg 74, Thr 75, Gln 77, Arg 97, Ile 98 and Asp 99 with a significant binding energy and disassociation constant.

EPA showed highest binding affinity with a binding energy of −3.67Kcal/mol and disassociation of 2.06mM among other PUFAs studied. Molecular interaction analysis showed polar and non-polar contacts with the binding cavity, which is shared by LXA4 involving the residues: Lys 18, Thr 20, Ala 21, Val 22, Phe 46, Glu 48, Lys 50, Arg 97, Ile 98 and Asp 99. Wherein, NPD1 showed lowest binding affinity for BDNF in comparison to other metabolites considered in the study.

Further, the present study reveals the common binding mode of PUFAs, metabolites and 4-MC to BDNF, inferred through molecular docking studies (Figure 3). Hence, the region of BDNF harbouring this common binding mode may be the probable site where small molecular enhancers bind, and thereby promoting neurotrophic activity.

Moreover, this site also does not interfere with the TrkB binding pockets in BDNF, which spans at the n-terminal region [36]. Hence, it could be hypothesized that the binding of PUFAs to BDNF shall exert allosteric effect on the TrkB binding pocket, thereby modulating the activity cascade. However, in vitro studies need to be performed to ascertain the in silico predictions.

The results of the present study are consistent with the hypothesis that PUFAs and their metabolites such as lipoxins, resolvins and protectins interact with BDNF and possibly, other neurotrophic factors and thus bring about their beneficial actions both in diabetes mellitus and neurological conditions such as depression [37-39]. Alternatively, the beneficial actions of BDNF in these clinical conditions could also be attributed to its interaction with PUFAs. Thus, a better understanding of the close interaction(s) between BDNF and PUFAs and their metabolites may pave way for the development of newer therapeutic strategies in diabetes mellitus, depression and other clinical conditions in which they are believed to play a significant role.

## Abbreviations

BDNF: Brain Derived Neurotrophic Factor; LXA4: Lipoxin A4; PUFAs: Poly Unsaturated Fatty Acids; 4-MC: 4-Methyl Catechol; AA: Arachidonic Acid; EPA: Eicosapentaneoic Acid; DHA: Docosahexanoic acid; NPD1: Neuroprotectin D1; RVE1: ResolvinE1; LA: Linoleic Acid; TrkB: Tropomyosin-Related Kinase B; Mc4r: melanocortin-4 receptor; HOMA: homeostasis model assessment; GROMACS: Groningen Machine for Chemical Simulations; TIP3P: Three-Point Transferable Intermolecular Potential; OPLS: Optimized Potentials for Liquid Simulations; PME: Particle Mesh Ewald; LGA: Lamarckian Genetic Algorithm; RMSD: Root Mean Square Deviation.

## Competing interests

The authors declare that they have no competing interests.

## Authors’ contributions

UV and SBR performed the bioinformatics and molecular docking studies. KK and JM performed the literature survey, helped in the bioinformatics studies. UND originated the idea and proposed the hypothesis that an interaction occurs between PUFAs and their metabolites and BDNF, initiated the bioinformatics studies and supervised the study. AN supervised and co-ordinated the study. All authors drafted the manuscript, read and approved the final version.
